# Supplementary material for: Multishell Diffusion MR Tractography Yields Morphological and Microstructural Information of the Anterior Optic Pathway: A Proof-of-Concept Study in Patients with Leber’s Hereditary Optic Neuropathy
Source: Int J Environ Res Public Health. 2022 Jun 5;19(11):6914. doi: 10.3390/ijerph19116914 (PMC9180110; doi:10.3390/ijerph19116914)
Supplement: Supplementary file 1 [file ijerph-19-06914-s001.zip › Supplementary Figure S1.pdf]

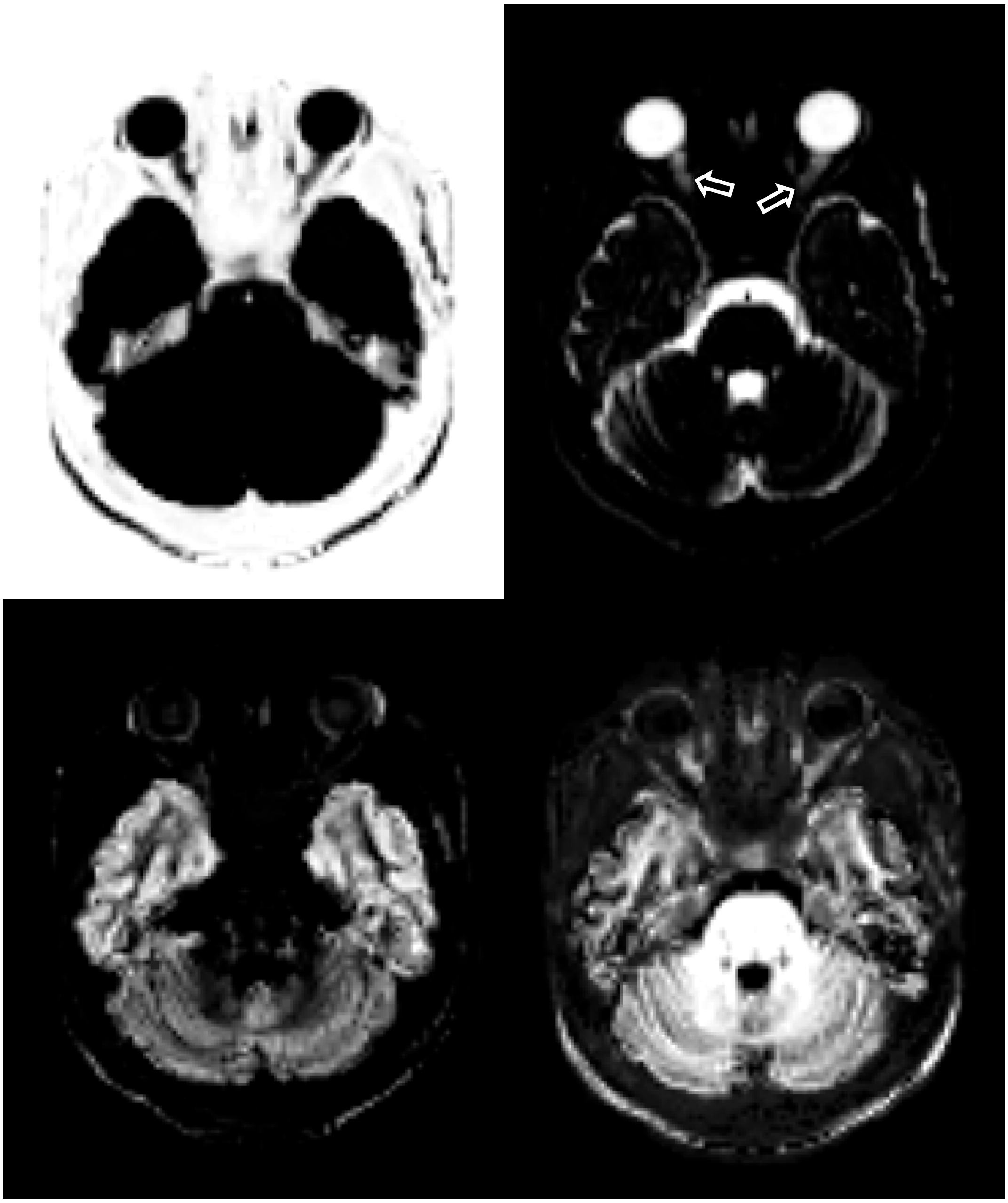

Figure S1. Signal fractions assigned to 3 tissue compartments by multi-tissue spherical deconvolution algorithm (msmt_csd) supplemented by background signal fraction, in one axial slice of a representative healthy control subject. (A) background; (B) CSF-like (normalized CSF, nCSF); (C) grey matter-like (nGM); (D) white matter-like (nWM). Note the variable fraction assigned to nGM and nCSF along the nerve, indicated by a small white arrow, and the absence of signal in the background component. For the purposes of visualization, the images have been interpolated to 1 mm resolution using a bicubic spline. The grey scale runs from 0% (black) to 90% (white).
