# Supplementary material for: Multishell Diffusion MR Tractography Yields Morphological and Microstructural Information of the Anterior Optic Pathway: A Proof-of-Concept Study in Patients with Leber’s Hereditary Optic Neuropathy
Source: Int J Environ Res Public Health. 2022 Jun 5;19(11):6914. doi: 10.3390/ijerph19116914 (PMC9180110; doi:10.3390/ijerph19116914)
Supplement: Supplementary file 1 [file ijerph-19-06914-s001.zip › Supplementary Tables.pdf]

Table S1. Morphometric analysis of the chiasm. Summary of morphometric assessment of the optic nerve and tract for each study population. The diameter of nerves (measured in mm) entering and leaving the chiasm was estimated by evaluation of volumetric T1-weighted images, as described in the text. Mean values of patient and healthy control groups were compared using the Student t-test.

	**LHON**		**HC**		**Comparison**
**Position**	**m**	**sd**	**m**	**sd**	**p(*t*)**
**Right anterior**	4.38	0.46	4.18	0.55	0.37
**Left anterior**	4.49	0.46	4.40	0.52	0.65
**Right posterior**	3.96	0.24	3.68	0.43	0.08
**Left posterior**	3.99	0.26	3.92	0.63	0.78

Note: HC: healthy control group. m: mean. s.d.: standard deviation. p(*t*): p-value of intergroup comparison

Table S2. Correlation between diffusion parameters and clinical/ophthalmological evaluation of patients within optic tract and optic nerve.

**Optic tract**		**FA**	**nGM**		**nWM**	
**Age (HC)**	Pearson's r	−0.356	0.081		−0.238	
	p-value	*0.074*	*0.693*		*0.242*	
**Age**	Pearson's r	−0.113	**0.681**	**	**−0.812**	***
	p-value	*0.677*	*0.004*		*<0.001*	
**Disease duration**	Pearson's r	0.328	0.028		−0.251	
	p-value	*0.215*	*0.919*		*0.348*	
**Visual acuity**	Pearson's r	−0.146	−0.164		0.363	
	p-value	*0.589*	*0.544*		*0.167*	
**Average RNFL**	Pearson's r	−0.209	−0.097		0.186	
	p-value	*0.438*	*0.721*		*0.490*	
**Optic nerve**		**FA**	**nGM**		**nWM**	
**Age (HC)**	Pearson's r	−0.142	**0.51**	**	0.343	
	p-value	*0.49*	*0.008*		*0.086*	
**Age**	Pearson's r	0.156	**0.517**	*	0.134	
	p-value	*0.563*	*0.04*		*0.62*	
**Disease duration**	Pearson's r	−0.089	−0.229		0.207	
	p-value	*0.744*	*0.395*		*0.443*	
**Visual acuity**	Pearson's r	−0.376	−0.281		**0.569**	*
	p-value	*0.151*	*0.291*		*0.021*	
**Average RNFL**	Pearson's r	0.449	0.299		**−0.576**	*
	p-value	*0.081*	*0.261*		*0.02*	

Note. * p < .05, ** p < .01, *** p < .001
FA: fractional anisotropy. nGM: normalized fraction of 'grey matter'-like signal. nGM: normalized fraction of 'white matter'-like signal. HC: healthy control group.
